# Cure rate in the elderly patients with diffuse large B cell lymphoma deteriorates after the age of 80—results from a single-center survey

**DOI:** 10.1007/s00277-021-04461-8

**Published:** 2021-02-25

**Authors:** Felix Freudenberger, Anke Ohler, Matthias Theobald, Georg Hess

**Affiliations:** Department of Internal Medicine III (Hematology, Oncology, Pneumology), University Medical Center of the Johannes Gutenberg University, Mainz, Germany

**Keywords:** Aggressive lymphoma, Geriatric patients, Treatment

## Abstract

The prognosis of elderly patients diagnosed with diffuse large B cell lymphoma (DLBCL) is considered to be clearly inferior to that of younger patients. Besides tumor biology and comorbidities, treatment selection due to an assumed reduced tolerability may contribute to this difference. With increasingly more patients diagnosed at advanced age, current treatment selections need to be reviewed carefully. Hence, we analyzed the results of patients above the age of 70 in whom a diagnosis of DLBCL was made. Whereas patients up to 80 were frequently selected for and were able to tolerate standard treatment (86% intended use, 74% completion), patients above the age of 80 years were not only treated more cautiously (67 and 60%, respectively) but did show inferior response to treatment with standard treatment (CR rate for intended R-CHOP use 64% vs. 43%). However, on an individual level, patients receiving and completing standard treatment obtained results that resemble the results of younger patients, irrespective if aged more than 80 and impose superior to prior reports in this age cohort. Median PFS for the entire group of patients was 3.44 years, with 4.83 years for patients below 80 and only 1.09 years for patients above the age of 80. The corresponding figures for OS were 7.38 years (estimated); after 2 years, OS was 81% in the younger cohort in contrast to 68% in patients > 80 years. However, for patients not planned to receive or not tolerating R-CHOP, results remain poor; tailored approaches for these patients are required.

## Introduction

Age is considered to be one of the most relevant risk factors for patients with cancer. In line with this general experience for patients with aggressive lymphoma, namely DLBCL, age is one of the contributing factors in the well-established International Prognostic Index (IPI) [1]. Treatment of DLBCL has evolved over recent decades, and the combination of rituximab to an anthracycline-containing regimen (CHOP and variations) is considered curative for the treatment of first-line patients [2]. Relative fractions of cure to this type of treatment correlate to different risk groups as defined by the IPI [3]. However, the definition of the IPI is based on data generated in the last century prior to the introduction of rituximab, and the impact of the further medical development over the last 20 years is not fully explored. Within the IPI, 60 years has been identified for discrimination into “young—and fit for treatment” or “old—and less fit for treatment”; however, for the majority of different treatment situations in oncology, this is not accepted as a treatment-limiting age border nowadays [4]. Today, the proportion of DLBCL patients not tolerating full-dose R-CHOP is considered to raise substantially over the age of 70, which is in line with current recommendations for prophylactic dose reductions of societies like ESMO, SIOG, or EORTC [5, 6]. Some authors even have suggested a negative impact of the use of full-dose chemo-immunotherapy, whereas others could not prove this finding [7, 8]. As premature dose reduction—solely based on age—may negatively impact overall survival, a critical review of current recommendations is indicated. As an example, in one of the few prospective investigations, using a dose-reduced R-CHOP (miniCHOP), after 1.7 years a PFS of only 47% was obtained, even after selecting for relatively fit patients [9]. Current retrospective data on elderly patients do show some limitations, as, e.g., heterogeneous histologies [10, 11], limited treatment information, or overrepresentation of specific subgroups [12]. To compare current results to these available data, we addressed the tolerability and efficacy to first-line treatment and outcome in all patients above the age of 70 years treated at our institution for DLBCL between 2003 and 2015 in the R-CHOP era.

## Patients and methods

### Patients

All patients at age 70 or older at the time of diagnosis registered in the electronic hospital chart system with a coding indicating DLBCL in the period from 2003 to 2015 were identified by a systematic electronic review. All patients were double-checked for correctness of diagnosis by second review of the pathology report in order to exclude cases with evidence of transformed indolent lymphoma or other aggressive lymphomas but DLBCL or any other conflicting result. Cell of origin genotyping was not available for the vast majority of patients at this time. Subsequently, a complete chart review was performed to analyze the entire disease course. This analysis was in accordance with regulations in place approved by the responsible ethics committee.

For evaluation of response—due to the heterogeneity of diagnostic standards over time—the Cheson (1999) classification was used as joint basis for all patients [13]. Progression was defined as the diagnosis of failure to respond to treatment, initiation of new treatment, disease progression or death—whatever came first. Overall survival was defined as time from diagnosis to death for whatever reason. Treatments were classified as follows: (R)-CHOP–like treatment was defined as any regiment containing at least an anthracycline, cyclophosphamide, and vincristine. If any dose reduction was planned already at the beginning of treatment, these treatments were summarized as mini-(R)-CHOP.

### Statistical analysis

Data were analyzed using GraphPad Prism version 8.0.2 for all explorative evaluations. Comparison of survival curves was done with Kaplan-Meier estimates, with a *p* of 0.05 formally used as discriminator. However, as this was a retrospective analysis, this was considered exploratory only.

## Results

### Patients

Initially, 344 patients were identified in the electronic system. After strict review, 136 patients fulfilled all criteria and were further followed for this analysis. Reasons for non-selection were: wrong entity, age, or date of initial diagnosis. The median observation time for patients alive at the time of analysis was 3.43 years. Relevant patient characteristics are summarized in Table 1. In brief, median age was 78 years (range 70–93 years), with an almost equal sex distribution (72 female versus 64 male patients). Two thirds of the patients were in between the age of 70 and 80 years; patients above the age of 80 represented 33% of the cases. All in all, approximately half of the patients had limited disease and extended disease, each. In respect to tumor manifestations, typical distribution of involvement was found (data not shown): 40% had cervical nodes, followed by abdominal (38%), thoracic (23%), and axillary manifestations (22%). Splenic involvement was found in 17%, followed by bone in 13%, liver in 12%, and skin manifestations in 6% of cases. The rate of involvement of bone marrow was rather low, with 7% of patients affected. B symptoms were present in 25% of patients; no B symptoms were present in 56% and not known in 18%. ECOG at diagnosis was evaluable in 2/3 of patients. Out of the evaluable patients, 68% had a good performance status of 0 or 1. LDH was elevated in 60% of patients, as was β2-microglobulin in 63%. Furthermore, anemia was present in 45% of all patients and lymphopenia in 40%; thrombocytopenia was rare (8%). Elevated creatinine levels as surrogate for partial or complete renal insufficiency—which could interfere with treatment fitness—were present in 20% of patients.

**Table 1:** Patient characteristics (available data)

**Characteristics**	70–80 y *n*=91 (67%)	>80 y *n*=45 (33%)	Statistics (<80 vs. >80)	Total *n*=136 (100%)
Stage at inclusion, *n* (%)				
I	23 (26%)	14 (32%)		37 (28%)
II	16 (18%)	13 (30%)		29 (22%)
III	14 (15%)	8 (18%)		22 (16%)
IV	37 (41%)	9 (20%)		46 (34%)
I/II	39 (43%)	27 (61%)	*p*= 0.49	66 (49%)
III/IV	51 (57%)	17 (39%)		68 (51%)
Nodal	40 (44%)	23 (51%)	*p*= 0.47	63 (46%)
Primarily extranodal	51 (56%)	22 (49%)		73 (54%)
B symptoms	24 (32%)	10 (28%)		34 (31%)
No B symptoms	51 (68%)	26 (72%)		77 (69%)
Pathology, *n* (%)				
BCL2^+^	32 (94%)	19 (79%)	*p*= 0.11	51 (88%)
BCL2^-^	2 (6%)	5 (21%)		7 (12%)
Ki67 <80%	29 (45%)	18 (49%)	*p*= 0.84	47 (47%)
Ki67 ≥80%	35 (55%)	19 (51%)		54 (53%)
Baseline ECOG performance status, *n* (%)				
0	20 (33%)	6 (20%)	*p*= 0.055 (ECOG 0/1 vs. >1)	26 (29%)
1	25 (42%)	10 (33%)		35 (39%)
>1	15 (25%)	14 (47%)		29 (32%)
Baseline IPI, *n* (%)				
0–1	18 (23%)	6 (15%)	*p*= 0.33 (IPI 0-2 vs. 3-5)	24 (20%)
2	22 (28%)	19 (48%)		41 (35%)
>2	38 (49%)	15 (37%)		53 (45%)
Lab results, *n* (%)				
β2-microglobulin ≤ 2.6mg/ml	29 (45%)	5 (18%)	*p*= 0.018	34 (37%)
β2-microglobulin > norm	35 (55%)	23 (82%)		58 (63%)
Creatinine ≤ 1	66 (85%)	29 (71%)	*p*= 0.09	95 (80%)
Creatinine > 1	12 (15%)	12 (29%)		24 (20%)

*IPI* International Prognostic Index, *BCL* B cell lymphoma

In evaluable patients, calculation of IPI revealed the following risk profile, which was well balanced between age groups: Low risk (0–1) was found in 20% of all patients, 23% in the age group 70–80 years and in 15% of patients older than 80 years. Low intermediate risk (2) was distributed among 35% of all patients, 28% of age group 70–80 years and 48% of patients older than 80 years. A high intermediate–/high-risk profile (3–5) was found in 45% of all patients, 49% of the 70–80 years cohort and 37% of the >80 years cohort (Table 1).

### Treatment

In most of the patients, R-CHOP–based treatment was chosen in curative intent (Table 2). Overall, 79% of all patients received anthracycline-based therapy: this was the case in 86% of patients between 70 and 80, but 67% for patients over the age of 80 years only. In the latter age group, 1/3 of patients were treated initially with palliative intent, in contrast to 14% in the younger age group. In total 28 patients did not receive R-CHOP–like treatment, 16 received palliative chemotherapy, 3 received radiotherapy only, and 9 patients were treated with BSC. There was no significant difference between female and male patients (78% vs. 80%). In terms of treatment adherence, 70% of patients could complete entire R-CHOP treatment. Reasons for early termination were toxicity in 14% and insufficient response in 6%. In further 6% of the patients treatment was terminated based on patient’s decision (data not shown). Putting age distribution into perspective, 74% of patients between 70 and 80 years could complete R-CHOP treatment as initially planned, whereas only 60% of the patients older than 80 years could do so. Therefore, 40% of the patients of age cohort >80 years, who started R-CHOP treatment, had to terminate the therapy prematurely (Table 2).

**Table 2:** First-line treatment and response to first-line treatment

Characteristics	Male *n*=64 (47%)	Female *n*=72 (53%)	70–80 y *n*=91 (67%)	>80 y *n*=45 (33%)	Statistics (<80 vs. >80)	Total *n*=136 (100%)
CHOP21 or equivalent	50 (78%)	58 (80%)	78 (86%)	30 (67%)	*p*= 0.01 (CHOP vs. palliative or no treatment) *p*=0.08 (CHOP started vs. CHOP completed)	108 (79%)
Completion of CHOP treatment, if intended	39 (78%)	37 (64%)	58 (74%)	18 (60%)		76 (70%)
Palliative or no treatment	14 (22%)	14 (19%)	13 (14%)	15 (33%)		28 (21%)
Response to first-line treatment, CHOP or equivalent, *n* (%)						
CR	26 (52%)	37 (64%)	50 (64%)	13 (43%)	*p*= 0.08 (response CR vs. <CR)	63 (58%)
PR	19 (38%)	12 (21%)	22 (28%)	9 (30%)		31 (29%)
SD or PD	5 (10%)	9 (16%)	6 (8%)	8(27%)		14 (13%)
PFS, median (years)	4.1	2.9	4.83	1.09		3.44
OS, median (years)	7.9	7.3	6.3	7.9		7.38

*CR*, complete response; *PR*, partial response; *SD*, stable disease; *PD*, progressive disease; *PFS*, progression-free survival; *OS*, overall survival; palliative regimen comprises rituximab, bendamustine (BR); rituximab, cyclophosphamide, vincristine, prednisone (R-CVP); rituximab, vincristine, prednisone (R-VIP), best supportive care (BSC)

A consolidative radiotherapy was performed in 21% of patients either due to initial bulky or extranodal disease or a residual mass after treatment. Twenty-two patients (78.6%) belong to the age group 70–80 years. Six patients (21.4%) belong to the age group > 80 years.

### Response to treatment

In those patients, in whom R-CHOP was initiated, 58% achieved a complete response (CR) and 29% a partial response (PR), whereas in 13% of patients, an inferior response was noted. There was a clear difference in response to treatment in between age cohorts—64% of patients up to 80 years achieved a CR in contrast to 43% of patients above the age of 80 (Table 2).

As expected, stage correlated with initial response to treatment: patients in stage I/II, who received treatment with R-CHOP, achieved a CR in 70% each. This decreased for stage III to 55% and to 45% for patients in stage IV. In patients with extranodal manifestations, CR rate was 54%. The correlation with the IPI of low risk (IPI 0–1), intermediate risk (IPI 2, 3), and high risk (IPI 4, 5) was as expected with 82, 52, and 35% achieving a CR, respectively (data not shown).

If we focus on the results in the small group of patients > 80 years, there was no difference in response according to stage (stage I/II, CR 35%, PR 30%, PD 35%; stage III/IV, CR 40%, PR 25%, PD 35%). However, in this population, extranodal manifestation correlated with extremely poor response rates (ORR 46%) with 54% being primary progressive or with early treatment discontinuation (data not shown).

### Progression-free survival

**Fig 1:**
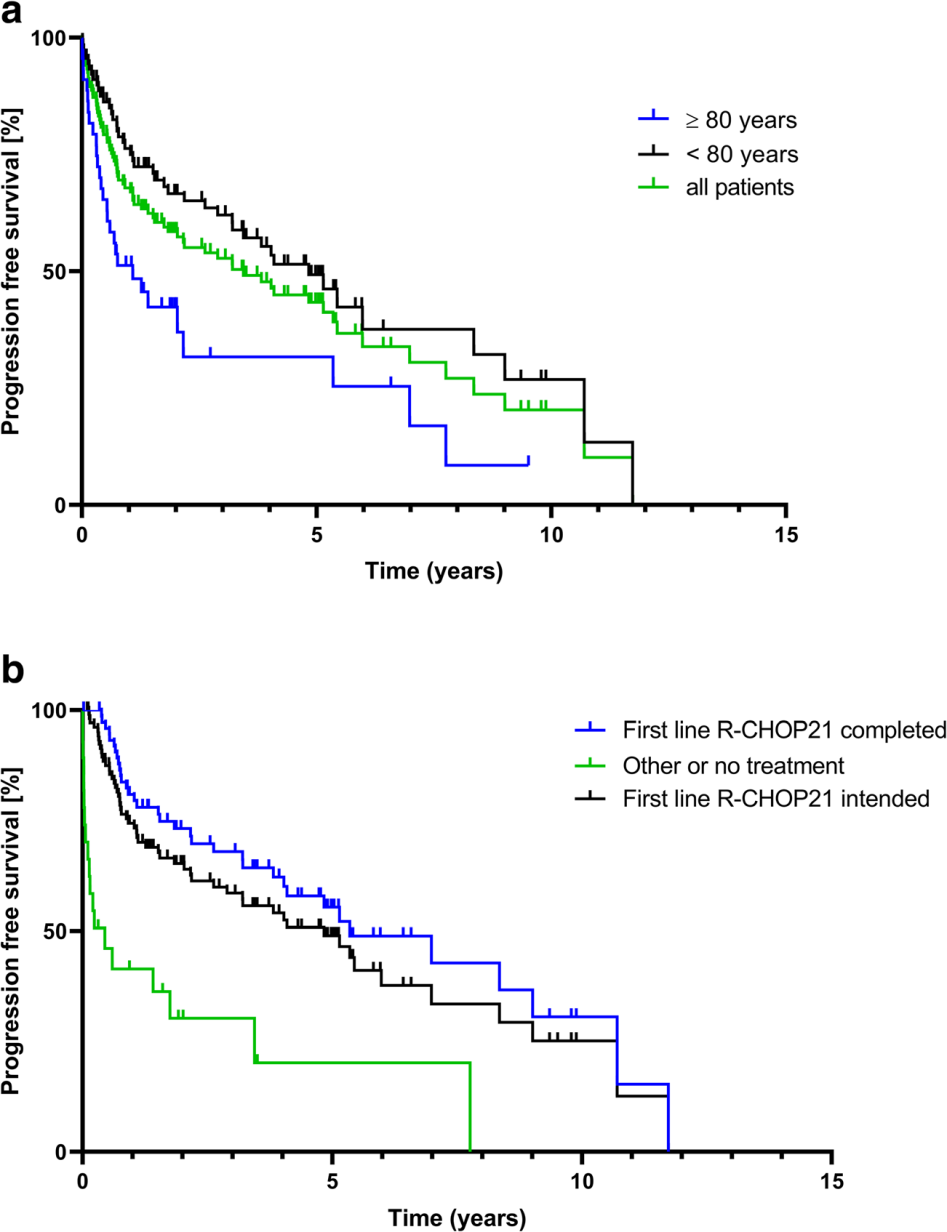
Kaplan-Meier curves of progression-free survival, results from time-to-event end points were analyzed according to Kaplan-Meier estimator. **a** PFS for the entire cohort and by age (*p*=0.0015 comparing < 80 years vs. ≥ 80 years; log-rank test) and **b** PFS in relation to first-line treatment and treatment adherence (*p*=<0.0001 comparing R-CHOP21 intended vs. other or no treatment; *p*=<0.0001 comparing R-CHOP21 completed vs. other or no treatment; log-rank test)

Median progression-free survival for the entire group of patients was 3.44 years; at 5 years, 43% of patients had no PFS defining event. Age was a significant risk factor for progression-free survival (*p*=0.0015). The median PFS of the age cohorts 70–80 years and ≥ 80 years were 4.83 years and 1.09 years, respectively (Fig. 1a).

Focusing on pre-therapeutic parameters, the following was found: there was no impact of sex; however, IPI correlated with the median progression-free survival; for IPI 1, 2/3, and 4/5, it was 5.44, 2.18, and 1.52 years, respectively. In detail, stage and ECOG had a major impact: e.g., patients with stage 1 had a median survival of 6 years vs. 1.84 years in stage IV. Interestingly, in patients with very good (ECOG 0) or good performance status (ECOG 1), median PFS was 6 and 5.14 years, whereas it was 1.4 years for patients with an ECOG of > 1. LDH proved to be discriminating, too (data not shown), as did parameters not included in the IPI, e.g., β2-MG (elevated vs. normal, median PFS 8.35 years vs. 2.89 years) and lymphopenia (5.44 vs. 2.18 years) (data not shown).

Looking further into the age cohort ≥ 80 years, the IPI had the following impact on PFS: low risk 5.35 years, intermediate risk 1.27 years, and high risk 0.54 years. Although elderly patients with low-risk IPI had a similar PFS compared to the entire low-risk IPI cohort, it was shown that patients older than 80 years had a shortened PFS with an intermediate- or high-risk IPI. Elevated LDH (0.6 years) vs. LDH normal (2.16 years), but no influence of β2-MG on PFS in the age group 80+ years (elevated 1.27 years vs. normal 1.41 years), was found in these patient cohorts. Performance status as a single risk factor resulted in a PFS of 7.8 years when ECOG was ≤ 1 and only 1.4 years when ECOG was >1 (data not shown).

Evaluation of choice and completion of treatment demonstrated major differences for all patients. We analyzed PFS in patients who started R-CHOP treatment (4.83 years) vs. patients who completed the entire R-CHOP regiment (5.35 years) vs. all patients with other or no treatment (0.45 years) (Fig. 1b). There was a clear correlation of CR with PFS (data not shown). Median time to relapse (from initial diagnosis) was less than half a year (0.45 years) considering patients older than 80 years, compared to 1 year (1.1 years) in the cohort 70–80 years.

### Overall survival

Typically for DLBCL, overall survival correlated well with progression-free survival. Estimated median OS for the entire group was 7.38 years with a median follow-up of 3.43 years for patients alive. In the different age cohorts, the following was noted: Estimated 5-year survival was 6.3 years for patients up to the age of 80, whereas it was 7.9 years in the patient aged 80 or more at the time of diagnosis, but follow-up was short.

Five years after diagnosis, 62% of patients remained alive. The IPI proved prognostic in our cohort: after 5 years, 83% of patients with IPI 1 remained alive in contrast to 57% for intermediate-risk and 31% for high-risk patients. Whereas LDH remained prognostic, the impact of β2-MG in PFS did not convert in OS differences (data not shown).

In the age cohort ≥ 80 years, the IPI had the following impact on OS: low risk 7.9 years vs. high risk 2.04 years. LDH as a single risk factor transferred into an estimated 5-year OS of 77% when in normal range and 57% when elevated. Performance status as a single risk factor did not convert in OS differences in the age cohort > 80 years (data not shown).

**Fig 2:**
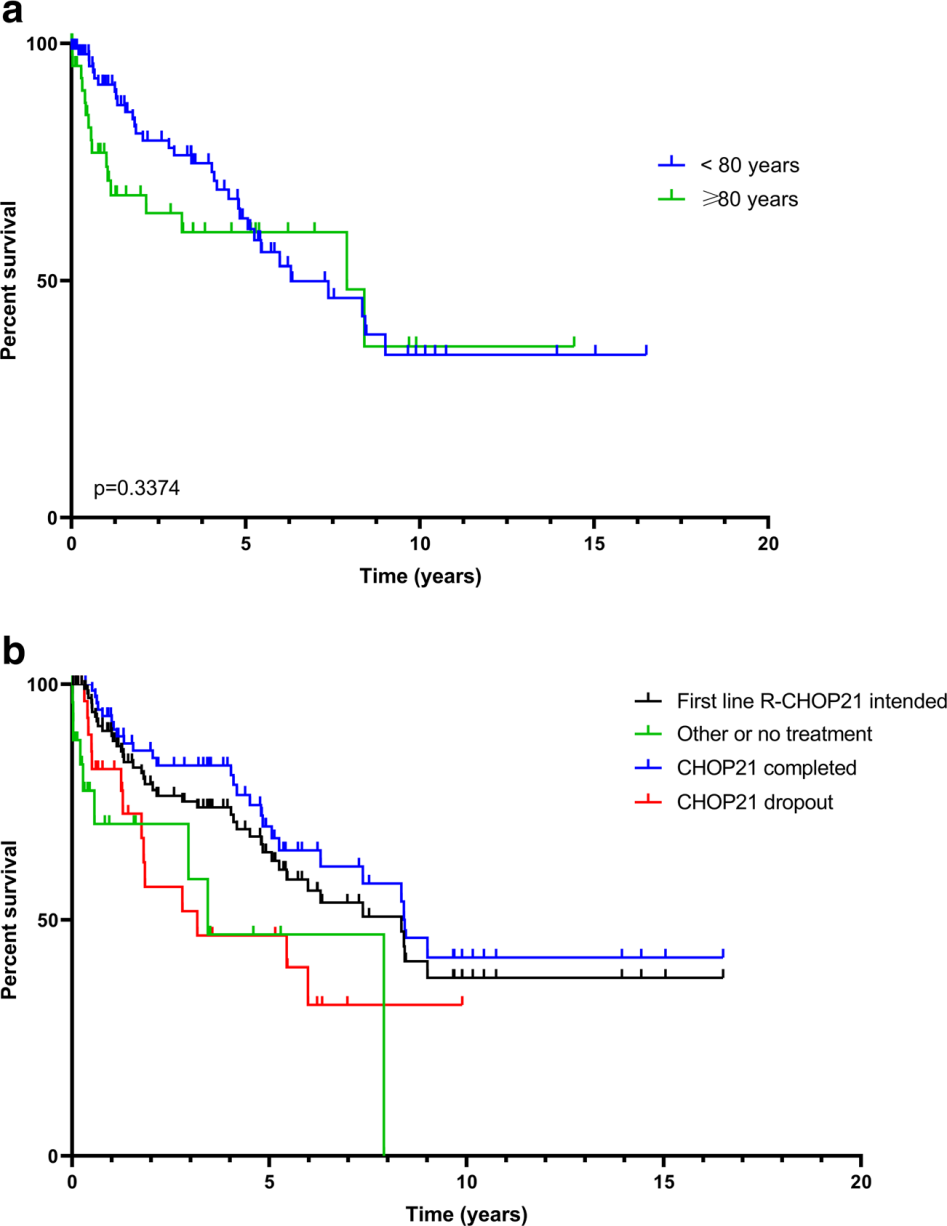
Kaplan-Meier curves of overall survival for all analyzed patients by age (**a**) and by first-line treatment and treatment adherence (**b**). Results from time-to-event end points were analyzed according to Kaplan-Meier estimator

Analyzing patients aged above 80 years, median follow-up was short (1.13 years). With this limitation in mind, after 1 year, OS was 74% in the age group 80+ years compared to 94% in the age group 70–80 years. After 2 years, OS was 68% as compared to the younger cohort with 81% (Fig. 2a).

Again, besides pre-therapeutic parameters, treatment choice was relevant: For all patients receiving R-CHOP, median OS was 8.35 years in contrast to 3.44 years for the entire group (Fig. 2b). Furthermore, response to first-line treatment showed a significant impact on median OS (CR vs. no CR *p*=0.0295, data not shown). As expected, time from relapse to death was short: for the entire group, it was 1.1 years, and for the age group ≥ 80 years, it was extremely short with 0.5 years.

Reasons of death were dominantly lymphoma associated (55%), as well as other reasons like preexisting illnesses (18%) and secondary malignancies (14%). However, for 10%, the cause of death remained unknown. Treatment-related death was noted in one of the patients (2%) only, due to pneumonia as a consequence of consolidative radiotherapy.

## Discussion

In the past, the age of 60 has been chosen as discriminator defining different treatment groups for patients with aggressive lymphoma [3]. This has been supported by data, where increased toxicity was found for elderly patients, e.g., for regimen like R-CHOP with addition of etoposide (R-CHOEP, dose-adjusted EPOCH) or, e.g., recently shown for the combination for R-CHOP with ibrutinib [14]. More profound side effects interacting with relative dose density and resulting in dose reductions were associated with impaired treatment results. On the other hand, treatments like high-dose therapy with autologous stem cell support or even CAR T cell therapy [15] have been shown to be feasible even in quite senior patients [16], if sufficiently fit for such a treatment. Although age continues to correlate with treatment tolerability, in any age group all efforts should be made to apply the best-balanced option in terms of efficacy and tolerability. In our analysis, we therefore addressed the question, if full-dose R-CHOP was feasible and effective in elderly and very elderly patients in a real-world scenario. Compared to controlled clinical trials, our cohort had a typical composition in terms of sex distribution, stage, and risk profile so that results can be considered representative [17]. As our institution is active in primary care of patients at any age and constitution, we would assume a limited selection bias—as compared to referral-only centers.

Interestingly, analyzing all subsequent patients above the age of 70 years and older treated in a defined period, it turned out that most of the patients had acceptable general performance status and full-dose treatment was feasible and effective. Treatment-related toxicity, especially death, was rather low in our series, as compared to that in others, e.g., the 8% observed in the series of Chihara [12] or others [7, 18], albeit no higher use of growth factors was noted. In addition, we were able to complete treatment to a higher extent as published in other series in all age groups analyzed [7, 8]. These differences in treatment-related mortality and the impact of comorbidities may be of special importance, when trying to understand the difference between series [19].

Especially patients aged 70–80 years showed results resembling younger patient groups. In contrast, there was more heterogeneity in the patients aged above 80 years: less patients were able to tolerate a full regimen, and if so, results were inferior. However, and importantly, if patients were able to tolerate, results remained positive, especially if there was limited stage, good ECOG, and other low-risk features. Interestingly, in our series the rate of patients intended to receive R-CHOP > 80 years (66%) was higher than in preceding periods [8, 10], which may reflect some change in treatment selection over time.

Overall survival for the younger patient group differed significantly from progression-free survival, indicating that relapse treatment was feasible to apply in these patients, in contrast to the very elderly. All in all, OS was promising, especially for low-risk patients. Our results compare favorable to other series especially for the very elderly (7.9 years for the entire cohort), but a limited follow-up needs to be kept in mind [8, 20]. Yet the 2-year OS rate (68%) of our elder age group (median age 83 years) seems to be plausible compared to a current multicentric, phase III, open-label, randomized trial conducted by the Lymphoma Study Association. In the study, 249 newly diagnosed patients aged at least 80 years (median age 83 years), treated with R-miniCHOP or R2-miniCHOP, showed a 2-year OS of approximately 66% [21]. As in other series, we found some negative impact of factors like lymphopenia or elevated LDH or extranodal manifestations in univariate analysis [22–25]. Importantly, completion of R-CHOP treatment directly impacted on OS, independent from age, which fits the results of other series [26].

A Swedish study group conducted a registry-based retrospective cohort study of all Swedish DLBCL patients diagnosed in 2000–2013, including more than 3500 patients older than 70 years [4]. Their data seem to confirm that our patient characteristics are representative. The patients show a similar distribution of stage, risk, and general condition. Surprisingly, less than one third of these patients were treated with a R-CHOP–like regiment, resulting in a comparably shorter overall survival, e.g., 1.2 years for all patients above the age of 80, which is not different from results in the pre-rituximab era [10, 20]. In this series however, only 21% of patients were definitely treated with R-CHOP–like treatment. If this is based on the real burden of comorbidities impacting on treatment selection or on reservation against more intensive treatment remains open but highlights short survival with a defensive treatment strategy. Chihara et al. evaluated 207 patients aged at least 80 years at the diagnosis of DLBCL from 2002 to 2014 at the MD Anderson Cancer Center [12]. Patients showed a slightly poorer risk distribution; nevertheless, they usually received a more intense therapy (R-CHOP 70%, R-EPOCH 6%, R-CEOP 6%). The 3-year PFS and OS rates were 55 and 54%, respectively. A total of 8% died as a result of treatment-related complications during the initial therapy. Interestingly our patients > 80 years reached a similar 3-year OS (60%) without any treatment-related deaths due to immunochemotherapy, which could indicate that a less dose-intense therapy is not inferior regarding overall outcome in elderly patients >80 years. Similar findings were published by a Danish study group, who identified 1011 DLBCL patients ≥75 years out of the Danish National Lymphoma Registry (LYFO), diagnosed from 2003 to 2012 [27]. Standard treatment (R-CHOP/CHOP–like) was initiated in 64%, ranging from 83% among patients aged 75–79 years to 32% among patient aged 85+ years. With standard treatment, median OS estimates were 4.6; 2.6, and 1.9 years for the age groups 75–79, 80–84, and 85+ years. Patients ≥ 80 years had similar OS regardless of intended R-CHOP dosing, whereas patients of 75–79 years scheduled for full dose had higher OS.

In consequence, our data underline the unmet medical need especially for the very elderly patients, if unable to tolerate treatment or in case of relapse. If treatments like polatuzumab will help to improve the prognosis of these patients need to be fully explored in the future [28]. This drug and other novel options like tafasitamab (in combination with lenalidomide) may be well-tolerated options widening the armamentarium for the very elderly, as typical side effect profiles of traditional salvage are present to a lesser extent [23–25]. In addition, biology-based classification, which in part correlates with age, may be of special importance for treatment selection, if targeted agents improve results for distinct subgroups [29].

Finally, our data show that dose interference or reduction for whatever reason is clearly associated with a dismal prognosis. Therefore, thorough evaluation of each individual treatment decision should be made to offer patients the treatment with the highest curative potential. As the majority of patients over the age of 70 years and approx. 40% of patients over 80 years do tolerate fully dosed treatment, age itself should not per se be considered as treatment-limiting criterion. However, for patients above the age of 80 years who cannot tolerate anthracycline-based regimen, treatment has to be considered palliative with currently available options. For these patients, optimization of first-line options integrating novel approaches as described above may be helpful, and such attempts are eagerly awaited.

## Data Availability

All materials are obtained at the Department of Hematology, Oncology and Pneumology at the University Medical School of the Johannes Gutenberg University.
